# Supplementary material for: Sex-specific retina–brain signatures link ERα/ERβ imbalance with gliosis in Alzheimer’s disease
Source: bioRxiv. 2026 May 5:2026.04.30.722000. Preprint. [Version 1] doi: 10.64898/2026.04.30.722000 (PMC13174345; doi:10.64898/2026.04.30.722000)
Supplement: Supplement 1 [file media-1.pdf]

Sex-specific retina–brain signatures links ERα/ERβ imbalance with gliosis in Alzheimer's disease

Saba Shahin, Jean-Philippe Vit, Altan Rentsendorj, Dieu-Trang Fuchs, Natalie Swerdlow, Bhakta Prasad Gaire, Edward Robinson, Anna Kissel, Larkin Hagerman, Ayzhah Williams, Alexander V. Ljubimov, Debra Hawes, Lon S. Schneider, Mehdi Mirzaei, Keith L. Black, Yosef Koronyo, Maya Koronyo-Hamaoui.

Table S1. List of human donors with demographic and neuropathological details whose brains and retinas were used in this study.
Table S2. List of human donors whose postmortem retinas were used for mass spectrometry and Western blot.
Table S3. List of human donors whose postmortem brains were used for mass spectrometry.
Table S4. List of antibodies for immunohistochemical and biochemical analyses.
Extended data Fig. 1: Sex-specific stratification of retinal and cerebral Aβ burden across disease stage, cognition, and APOEε4 genotype.
Extended data Fig. 2: Sex-specific stratification of retinal and cerebral pathological tau burden across disease stage, cognition, and APOEε4 genotype.
Extended data Fig. 3: Sex-specific associations between retinal $A\beta_{42}$/tau burden, cerebral pathology, disease stage, and cognition.
Extended data Fig. 4: Sex-specific associations between neurodegeneration, cerebral pathology, disease stage, and cognition.
Extended data Fig. 5: Sex-specific stratification and associations of retinal and cerebral gliosis across neuropathological burden, disease stages and cognitive status.
Extended data Fig. 6: Expression of ER-α in the cerebral cortex of NC, MCI and AD individuals.
Extended data Fig. 7: Expression of ER-α in the retina of NC, MCI and AD individuals
Extended data Fig. 8: Expression of ER-β in the cerebral cortex of NC, MCI and AD individuals.
Extended data Fig. 9: Expression of ER-β in the retina of NC, MCI and AD individuals.
Extended data Fig. 10: Sex-specific associations of retinal and cerebral ER-α with disease stage, cognition, and *APOEε4* genotype.
Extended data Fig. 11: Sex-specific associations of retinal and cerebral ER-β with disease stage, cognition, and *APOEε4* genotype.

Table S1. List of human donors with demographic and neuropathological details whose brains and retinas were used in this study.

Diagnosis	Age (years)	Sex	PMI (hours)	APOE	Thal-A	Braak- B	CERAD- C	Analysis
NC1	93	F	12.00	e3/e2	3	2	3	IHC
NC2	85	F	4.50	e3/e3	2	1	2	IHC
NC3	99	F	21.00	e3/e3	1	2	1	IHC
NC4	95	F	7.25	e3/e3	3	3	2	IHC
NC5	95	F	3.15	n.a.	1	0	0	IHC
NC6	92	F	7.00	n.a.	1	1	0	IHC
NC7	97	F	31.00	n.a.	3	1	2	IHC
NC8	102	F	37.00	n.a.	3	1	3	IHC
NC9	100	F	21.50	e3/e4	2	2	3	IHC
NC10	98	F	21.00	e3/e3	2	1	2	IHC
NC11	95	F	5.30	e3/e3	0	2	0	IHC
NC12	91	F	22.00	n.a.	2	2	0.5	IHC
NC13	93	F	5.60	n.a.	n.a.	n.a.	n.a.	IHC
NC14	58	F	5.20	n.a.	n.a.	n.a.	n.a.	IHC
NC15	88	F	5.40	n.a.	n.a.	n.a.	n.a.	IHC
NC16	86	F	5.00	n.a.	n.a.	n.a.	n.a.	IHC
NC17	76	F	3.60	n.a.	n.a.	n.a.	n.a.	IHC
NC18	75	F	5.20	n.a.	n.a.	n.a.	n.a.	IHC
NC19	72	F	5.53	n.a.	n.a.	n.a.	n.a.	IHC/WB
NC20	76	F	7.30	n.a.	n.a.	n.a.	n.a.	IHC
NC21	77	F	5.70	n.a.	n.a.	n.a.	n.a.	IHC
NC22	71	F	7.30	n.a.	n.a.	n.a.	n.a.	IHC
NC23	73	F	12.70	n.a.	n.a.	n.a.	n.a.	IHC
NC24	70	F	n.a.	n.a.	n.a.	n.a.	n.a.	IHC
NC25	81	M	7.24	e3/e4	3	1	2	IHC/WB
NC26	95	M	6.50	e3/e3	1	1	1	IHC/WB
NC27	76	M	11.25	e3/e3	2	0	2	IHC
NC28	80	M	n.a.	n.a.	n.a.	n.a.	n.a.	IHC
NC29	58	M	n.a.	n.a.	n.a.	n.a.	n.a.	IHC
NC30	69	M	6.15	n.a.	0	0	1	IHC
NC31	88	M	5.00	n.a.	2	1	2	IHC
NC32	61	M	6.00	n.a.	0	0	0	IHC
NC33	88	M	19.00	e3/e4	2	3	2	IHC
NC34	77	M	5.20	n.a.	n.a.	n.a.	n.a.	IHC
NC35	84	M	8.30	n.a.	n.a.	n.a.	n.a.	IHC
NC36	70	M	7.20	n.a.	n.a.	n.a.	n.a.	IHC
NC37	74	M	N/A	n.a.	n.a.	n.a.	n.a.	IHC
NC38	87	M	7.90	n.a.	n.a.	n.a.	n.a.	IHC
NC39	78	M	6.50	n.a.	n.a.	n.a.	n.a.	IHC
NC40	73	M	9.40	n.a.	n.a.	n.a.	n.a.	IHC
NC41	75	M	6.30	n.a.	n.a.	n.a.	n.a.	IHC
NC42	66	M	na	n.a.	n.a.	n.a.	n.a.	IHC
MCI1	86	F	18.00	e3/e4	3	1	3	IHC
MCI2	93	F	7.75	e3/e3	3	2	2	IHC

Diagnosis	Age	Sex	PMI	APOE	Thal-A	Braak- B	CERAD- C	Analysis
MCI3	94	F	10.50	e3/e3	2	1	2	IHC
MCI4	89	F	1.50	e3/e3	1	2	2	IHC
MCI5	91	F	4.45	n.a.	2	2	2	IHC
MCI6	98	F	7.50	n.a.	2	3	2	IHC
MCI7	87	F	4.00	e3/e3	3	3	3	IHC
MCI8	80	F	3.75	n.a..	3	3	3	IHC
MCI9	88	F	18.00	e3/e3	2	3	3	IHC
MCI10	93	M	12.50	e3/e2	2	0	2	IHC
MCI11	97	M	3.50	e3/e3	2	3	3	IHC
MCI12	83	M	3.00	n.a.	2	3	2	IHC
MCI13	88	M	12.00	n.a.	1	2	2	IHC
MCI14	80	M	9.00	e3/e3	3	3	2	IHC
MCI15	85	M	16.50	n.a.	0	1	3	IHC
MCI16	75	M	45.00	n.a.	1	1	2	IHC
MCI17	90	M	28.25	e4/e2	1	1	1	IHC
MCI18	99	M	6.70	e3/e3	3	2	3	IHC
AD1	48	F	4.00	n.a.	3	3	3	IHC/WB
AD2	42	F	30.00	e3/e3	3	3	3	IHC
AD3	51	F	6.00	n.a.	3	3	3	IHC
AD4	90	F	9.00	n.a.	2	3	3	IHC
AD5	100	F	7.00	n.a.	2	3	3	IHC
AD6	87	F	10.00	e3/e4	2	3	3	IHC
AD7	70	F	6.25	n.a.	3	3	3	IHC
AD8	66	F	17.00	e3/e3	3	3	3	IHC
AD9	99	F	4.50	e3/e3	3	2	3	IHC
AD10	85	F	8.50	e3/e3	3	3	3	IHC
AD11	81	F	3.00	n.a.	3	3	3	IHC
AD12	92	F	11.25	e3/e3	2	3	2	IHC
AD13	88	F	5.00	n.a.	2	3	3	IHC
AD14	86	F	6.00	e3/e4	3	3	2	IHC
AD15	93	F	12.00	n.a.	2	2	2	IHC
AD16	94	F	5.50	e3/e3	3	3	3	IHC/WB
AD17	81	F	7.50	e3/e3	3	3	3	IHC
AD18	90	F	19.00	e3/e4	3	3	3	IHC
AD19	93	F	5.50	n.a.	2	3	3	IHC
AD20	87	F	4.50	e3/e4	3	3	3	IHC
AD21	81	F	4.50	n.a.	3	3	3	IHC
AD22	81	F	5.00	n.a.	3	3	3	IHC/WB
AD23	88	F	5.25	e3/e4	3	3	3	IHC
AD24	71	F	3.50	n.a.	3	3	3	IHC
AD25	76	F	18.25	e3/e4	3	3	3	IHC
AD26	96	F	9.00	n.a.	1	1	3	IHC
AD27	93	F	9.00	e3/e3	3	2	2	IHC
AD28	91	F	35.25	e3/e2	3	3	3	IHC
AD29	96	F	52.00	e3/e3	3	3	3	IHC
AD30	88	F	25.00	n.a.	2	3	3	IHC

Diagnosis	Age	Sex	PMI	APOE	Thal-A	Braak- B	CERAD- C	Analysis
AD31	93	F	4.00	n.a.	3	3	3	IHC
AD32	80	F	33.50	e4/e2	3	3	3	IHC
AD33	87	F	12.50	e3/e3	3	3	3	IHC
AD34	87	F	31.00	e3/e3	3	3	3	IHC
AD35	85	F	32.50	e3/e3	2	3	3	IHC
AD36	100	F	24.00	e3/e3	3	3	3	IHC
AD37	64	F	3.38	n.a.	3	3	3	IHC
AD38	89	F	9.00	n.a.	3	3	3	IHC
AD39	97	F	9.16	n.a.	3	3	3	IHC
AD40	90	F	5.00	n.a.	3	3	3	IHC
AD41	63	F	6.50	n.a.	3	3	3	IHC
AD42	94	F	5.25	n.a.	3	3	3	IHC
AD43	90	F	3.75	n.a.	3	3	3	IHC
AD44	70	F	5.00	e4/e4	2	2	2	IHC
AD45	65	F	3.00	n.a.	3	3	3	IHC
AD46	85	F	4.00	n.a.	2	3	3	IHC
AD47	74	F	n.a.	n.a.	n.a.	n.a.	n.a.	IHC
AD48	39	M	8.00	n.a.	3	3	3	IHC
AD49	40	M	7.50	e3/e3	3	2	3	IHC
AD50	90	M	19.00	n.a.	3	3	3	IHC
AD51	88	M	7.50	e3/e4	2	3	2	IHC/WB
AD52	77	M	8.25	e3/e4	3	3	3	IHC
AD53	81	M	6.50	e4/e4	3	3	3	IHC
AD54	83	M	6.00	n.a.	3	2	3	IHC
AD55	65	M	6.75	e4/e4	3	3	3	IHC
AD56	66	M	11.75	n.a.	3	3	3	IHC
AD57	88	M	7.50	e3/e2	3	3	3	IHC
AD58	90	M	7.00	e3/e4	3	2	3	IHC
AD59	79	M	6.50	n.a.	3	3	3	IHC
AD60	88	M	4.50	n.a.	2	2	3	IHC
AD61	97	M	5.25	e3/e3	3	2	3	IHC
AD62	92	M	8.50	e3/e3	3	3	3	IHC
AD63	79	M	6.00	n.a.	2	3	2	IHC
AD64	90	M	4.00	n.a.	3	3	2	IHC
AD65	75	M	9.67	n.a.	3	3	3	IHC
AD66	78	M	10.83	e3/e4	3	3	3	IHC
AD67	85	M	4.33	n.a.	3	2	3	IHC
AD68	69	M	13.00	e3/e4	3	3	3	IHC
AD69	83	M	17.00	e3/e3	3	3	3	IHC
AD70	88	M	72.00	e3/e3	3	3	3	IHC
AD71	65	M	62.00	e3/e4	3	3	3	IHC
AD72	86	M	31.50	e4/e4	3	3	3	IHC
AD73	96	M	7.00	e3/e3	3	3	3	IHC
AD74	99	M	17.75	n.a.	3	3	3	IHC
AD75	94	M	20.00	e3/e3	1	2	1	IHC
AD76	63	M	23.50	e3/e4	3	3	2.5	IHC

Diagnosis	Age	Sex	PMI	APOE	Thal-A	Braak- B	CERAD- C	Analysis
AD77	86	M	15.75	e3/e4	3	2	2	IHC
AD78	59	M	15.00	n.a.	3	3	2	IHC
AD79	72	M	7.00	e3/e4	3	1	3	IHC
AD80	78	M	8.25	e3/e4	3	2	3	IHC
AD81	65	M	5.00	n.a.	3	3	3	IHC
AD82	83	M	13.00	n.a.	3	3	3	IHC
AD83	71	M	13.67	n.a.	3	3	3	IHC
AD84	87	M	4.50	e3/e4	3	3	2	IHC
AD85	78	M	9.50	n.a.	3	3	3	IHC
AD86	70	M	4.00	e3/e3	3	3	3	IHC
AD87	86	M	4.00	n.a.	3	3	3	IHC
AD88	69	M	2.50	n.a.	3	3	3	IHC
AD89	85	M	4.83	n.a.	n.a.	n.a.	n.a.	IHC
AD90	79	M	10.50	n.a.	n.a.	n.a.	n.a.	IHC
AD91	59	M	7.00	n.a.	n.a.	n.a.	n.a.	IHC
AD92	72	M	n.a.	n.a.	n.a.	n.a.	n.a.	IHC

A (Thal), Aβ plaque score modified from Thal; B (Braak), NFT stage modified from Braak; C (CERAD), neuritic plaque score modified from CERAD.

Abbreviations: AD, Alzheimer's disease dementia; APOE, apolipoprotein; IHC, immunohistology; MCI, Mild cognitive impairment; n.a., not available NC, normal cognition; PMI, post-mortem interval; WB, Western blot.

Table S2. List of human donors whose postmortem retinas were used for mass spectrometry and Western blot.

Donor	Sex	Age at Death	Thal (A)	Braak (B)	CERAD (C)	Braak Stage	APOE Status	Analysis
AD1	F	48	3	3	3	5.5	n.a.	MS
AD2	F	93	2	3	3	5	n.a.	MS
AD3	M	88	2	3	2	5.5	e3/e4	MS
AD4	M	88	1	2	2	3	n.a.	MS
AD5	F	94	3	3	3	5.5	e3/e3	MS
AD6	F	100	2	3	3	5.5	n.a.	MS
AD7	F	74	n.a.	n.a.	n.a.	n.a.	n.a.	WB
NC1	M	81	3	1	2	1.5	e3/e4	MS
NC2	F	75	n.a.	n.a.	n.a.	n.a.	n.a.	MS/WB
NC3	F	72	n.a.	n.a.	n.a.	n.a.	n.a.	MS
NC4	M	69	n.a.	n.a.	n.a.	n.a.	n.a.	MS/WB
NC5	F	79	n.a.	n.a.	n.a.	n.a.	n.a.	MS/WB
NC6	M	85	0	1	3	1.5	n.a.	MS

A (Thal), Aβ plaque score modified from Thal; B (Braak), NFT stage modified from Braak; C (CERAD), neuritic plaque score modified from CERAD.
Abbreviations: AD, Alzheimer's disease dementia; APOE, apolipoprotein; IHC, immunohistology; MCI, Mild cognitive impairment; n.a., not available NC, normal cognition.

Table S3. List of human donors whose postmortem brains were used for mass spectrometry.

Donor	Sex	Age at Death	Plaque Stage	Braak Stage	Neuritic Plaque	APOE Status
AD1	M	86	C	6	4	e3/e4
AD2	F	89	C	6	4	e3/e4
AD3	F	95	C	6	3	e2/e3
AD4	F	93	C	6	4	e3/e3
AD5	F	88	C	5	4	e3/e3
AD6	M	92	C	5	4	e3/e3
AD7	M	86	C	5	4	e3/e3
AD8	F	98	C	6	4	e3/e3
AD9	F	91	C	6	4	e3/e3
AD10	F	82	C	6	4	e3/e3
NC1	F	87	0	2	1	e2/e3
NC2	F	91	0	4	1	e2/e3
NC3	F	95	0	2	1	e3/e3
NC4	F	90	B	3	3	e3/e3
NC5	M	96	0	2	1	e2/e3
NC6	M	94	0	1	1	e3/e3
NC7	M	86	0	2	2	e3/e3
NC8	F	91	A	2	2	e3/e3

Plaque Stage: 0, none; A, phase I-II; B, phase III; C, phase IV-V.

Neuritic Plaque scores: 4, frequent NP; 3, moderate NP; 2, sparce NP; 1, no NPs.

Abbreviations: AD, Alzheimer's disease; APOE, apolipoprotein; NC, normal cognition. The original human brain and retinal mass spectrometry data on this cohort were previously published in Koronyo et al., Acta Neuropathologica 2023[1].

Table S4. List of antibodies for immunohistochemical and biochemical analyses.

Antibodies	Source species	Target species	Dilution	Application	Source	Catalog #
**Primary Antibodies**						
GFAP pAb	Goat	Hu, ms	1:500	IHC-F	Invitrogen	13-0300
IBA1 mAb	Rabbit	Hu, ms	1:400	IHC-F	Wako	019-19741
IBA1 mAb	Goat	Hu, ms	1:300	IHC-F	NovusBio	NB100-1028
Aβ_{42} (12F4) mAb	Mouse	Hu	1:1000	WB	Biolegend	805501
Aβ_{40} (11A50-B10) mAb	Mouse	Hu	1:200	IHC-F	Biolegend	805401
Tau Oligomers (T22) mAb	Rabbit	Hu	1:200	IHC-F	Dr. Rakez Kayed	-
Phospho-tau (Ser396) pAb	Rabbit	Hu	1:500	IHC-F	Anaspec	AS-54977
MC-1	Mouse	Hu	1:200	IHC-F	Dr. Peter Davies	-
Claudin-5 mAb	Mouse	Hu, ms	1:20	IHC-F	ThermoFisher	35-2500
Zonula Occluden-1 (ZO1) pAb	Rabbit	Hu, ms	1:50	IHC-F	ThermoFisher	61-7300
ER-α mAb	Mouse	Hu, ms	1:100-1:1000	IHC/WB	ThermoFisher	MA1-80216
ER-β pAb	Rabbit	Hu, ms	1:100-1:1000	IHC/WB	ThermoFisher	PA1-313
GAPDH (D16H11) mAb	Rabbit	Hu, ms	1:1000	WB	Cell signaling	5174
GAPDH mAb	Mouse	Hu, ms	1:1000	WB	Millipore Sigma	G8795
**Secondary Antibodies**						
Cy3 (anti-mouse, -rat, -goat, & -rabbit)	Donkey		1:200	IF	Jackson ImmunoResearch Laboratories	715-165-150, 112-165-167, 705-165-147, 711-165-152
Cy5 (anti-mouse, -rat, -goat, & -rabbit)	Donkey		1:200	IF	Jackson ImmunoResearch Laboratories	715-175-150, 712-175-153, 705-175-147, 711-175-152
Cy2 (anti-goat, & -rabbit)	Donkey		1:200	IF	Jackson ImmunoResearch Laboratories	705-225-147, 711-225-152
IRDye® 680RD pAb	Rabbit	Hu, ms	1:10000	WB	Licorbio	926-68071
IRDye® 800CW pAb	Rabbit	Hu, ms	1:10000	WB	Licorbio	926-32211
IRDye® 680RD pAb	Mouse	Hu, ms	1:10000	WB	Licorbio	926-68070
IRDye® 800CW pAb	Mouse	Hu, ms	1:10000	WB	Licorbio	926-32210

Abbreviations: Aβ - amyloid β-protein; GFAP - glial fibrillary acidic protein; IBA1 - ionized calcium binding adaptor molecule 1; IHC - immunohistochemistry; IHC-F - fluorescence; Hu - human; ms - mouse; mAb - monoclonal antibody; pAb - polyclonal antibody. WB - Western blot.

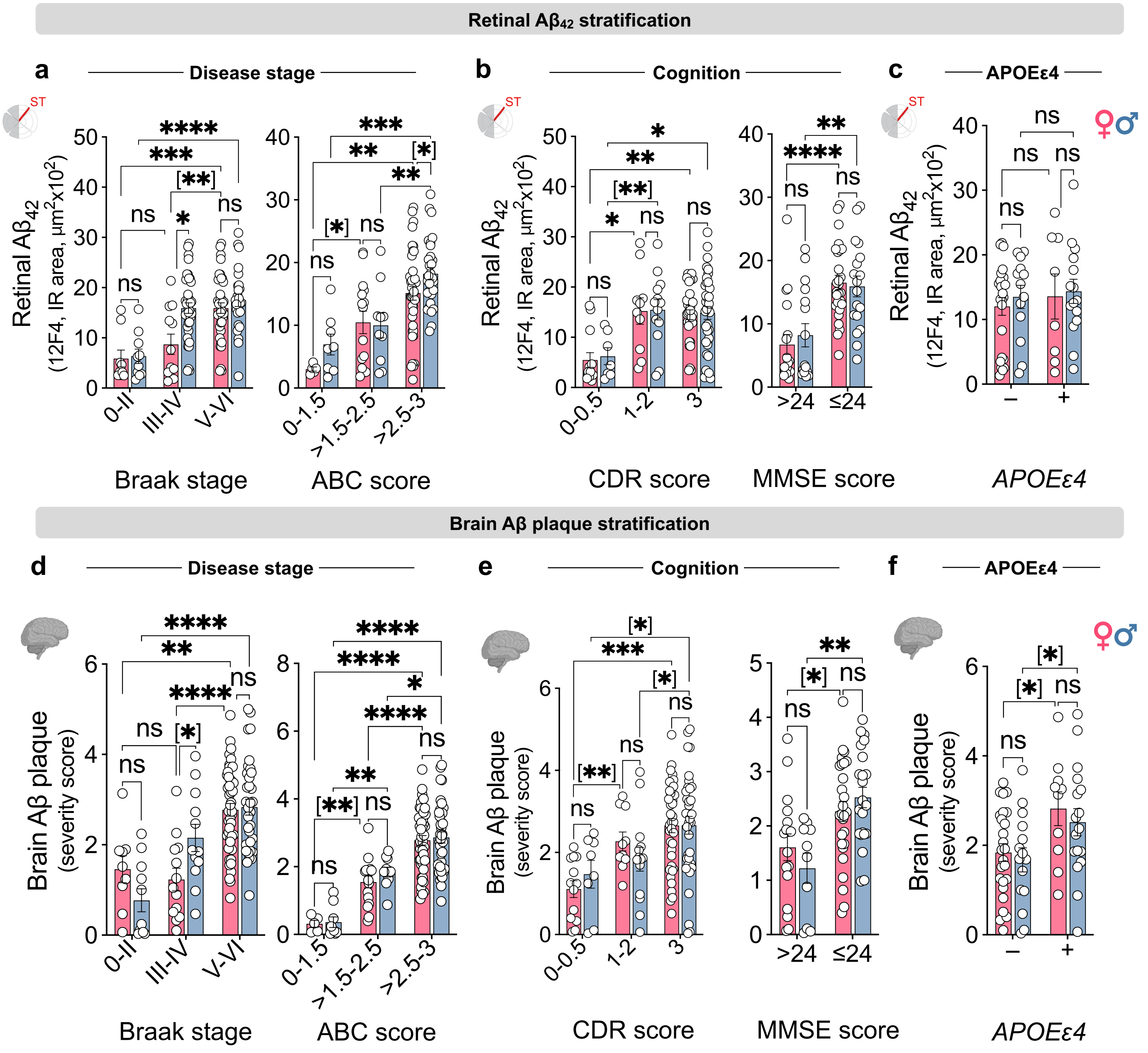

Extended data Fig. 1: Sex-specific stratification of retinal and cerebral Aβ burden across disease stage, cognition, and *APOEε4* genotype. a-c Retinal 12F4$^+$ $Aβ_{42}$ immunoreactivity (IR) area in females and males stratified by **a** Braak stage (n = 9F/10M, 0–II; 12F/12M, III–IV; 38F/26M, V–VI) and ABC score (n = 5F/8M, 0–1.5; 15F/10M, >1.5–2.5; 39F/30M, >2.5–3) **b** cognitive status based on CDR (n = 13F/7M, 0-0.5; 13F/10M, 1-2; 31F/36M, 3) and MMSE score (n = 18F/16M, >24; 27F/17M, ≤24) and **c** *APOEε4* genotype (n = 8F/15M ε4 carriers; 24F/13M, non-carriers). **d-f** Cerebral Aβ burden in females and males stratified by **d** Braak stage (n = 9F/11M, 0–II; 13F/12M, III–IV; 45F/34M, V–VI) and ABC score (n = 5F/9M, 0–1.5; 16F/10M, >1.5–2.5; 46F/38M, >2.5–3) **e** CDR (n = 13F/8M, 0-0.5; 10F/14M, 1-2; 38F/35M, 3) and MMSE score (n = 17F/12M, >24; 27F/20M, ≤24) and **f** *APOEε4* genotype (n = 10F/17M ε4 carriers; 25F/15M, non-carriers). Data are presented as individual subjects (circles), and group means ± SEMs. Statistical analyses used 2-way ANOVA with Bonferroni's post hoc tests for 3- or more group comparisons and 2-tailed unpaired Student's *t*-test for 2-group comparisons (in parentheses). **P* ≤ 0.05, ***P* ≤ 0.01, ****P* ≤ 0.001, *****P* ≤ 0.0001, ns: nonsignificant.*

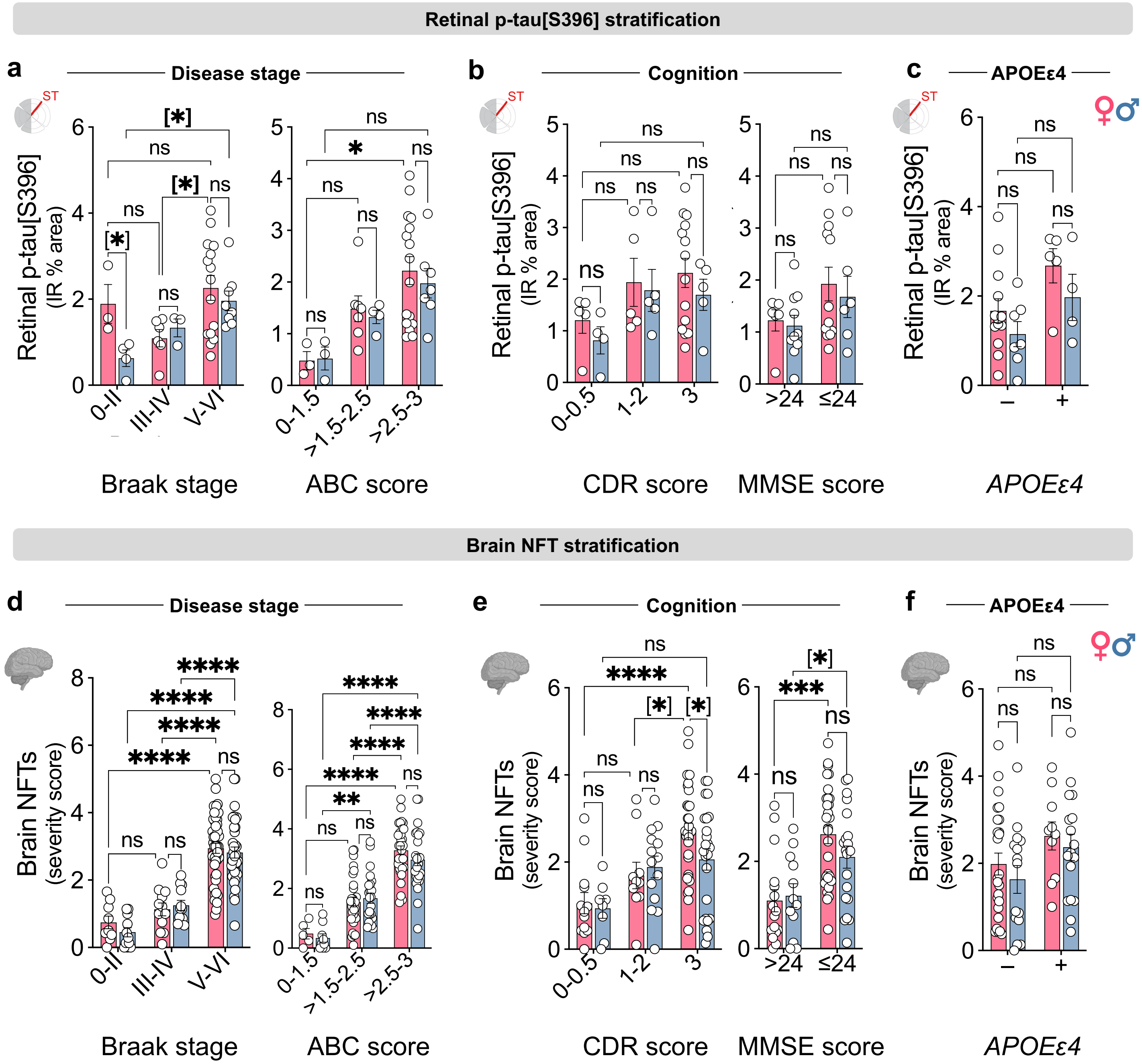

Extended data Fig. 2: Sex-specific stratification of retinal and cerebral pathological tau burden across disease stage, cognition, and *APOEε4* genotype. a-c Retinal p-tau[S396] immunoreactivity (IR) percentage area in females and males stratified by **a** Braak stage (n = 3F/4M, 0–II; 6F/3M, III–IV; 15F/8M, V–VI) and ABC score (n = 3F/3M, 0–1.5; 7F/4M, >1.5–2.5; 16F/7M, >2.5–3) **b** CDR (n = 5F/4M, 0-0.5; 5F/5M, 1-2; 14F/5M, 3) and MMSE scores (n = 6F/10M, >24; 12F/6M, ≤24) and **c** *APOEε4* genotype (n = 5F/4M ε4 carriers; 13F/7M, non-carriers). **d-f** Cerebral NFTs burden in females and males stratified by **d** Braak stage (n = 9F/11M, 0–II; 12F/12M, III–IV; 46F/34M, V–VI) and ABC score (n = 5F/9M, 0–1.5; 29F/23M, >1.5–2.5; 33F/25M, >2.5–3) **e** CDR (n = 13F/8M, 0-0.5; 10F/14M, 1-2; 31F/24M, 3) and MMSE score (n = 16F/12M, >24; 29F/20M, ≤24) and **f** *APOEε4* genotype (n = 10F/17M ε4 carriers; 25F/15M, non-carriers). Data are presented as individual subjects (circles), and group means ± SEMs. Statistical analyses used 2-way ANOVA with Bonferroni's post hoc tests for 3- or more group comparisons and 2-tailed unpaired Student's *t*-test for 2-group comparisons (in parentheses). **P ≤ 0.05, **P ≤ 0.01, ***P ≤ 0.001, ****P ≤ 0.0001, ns: nonsignificant.*

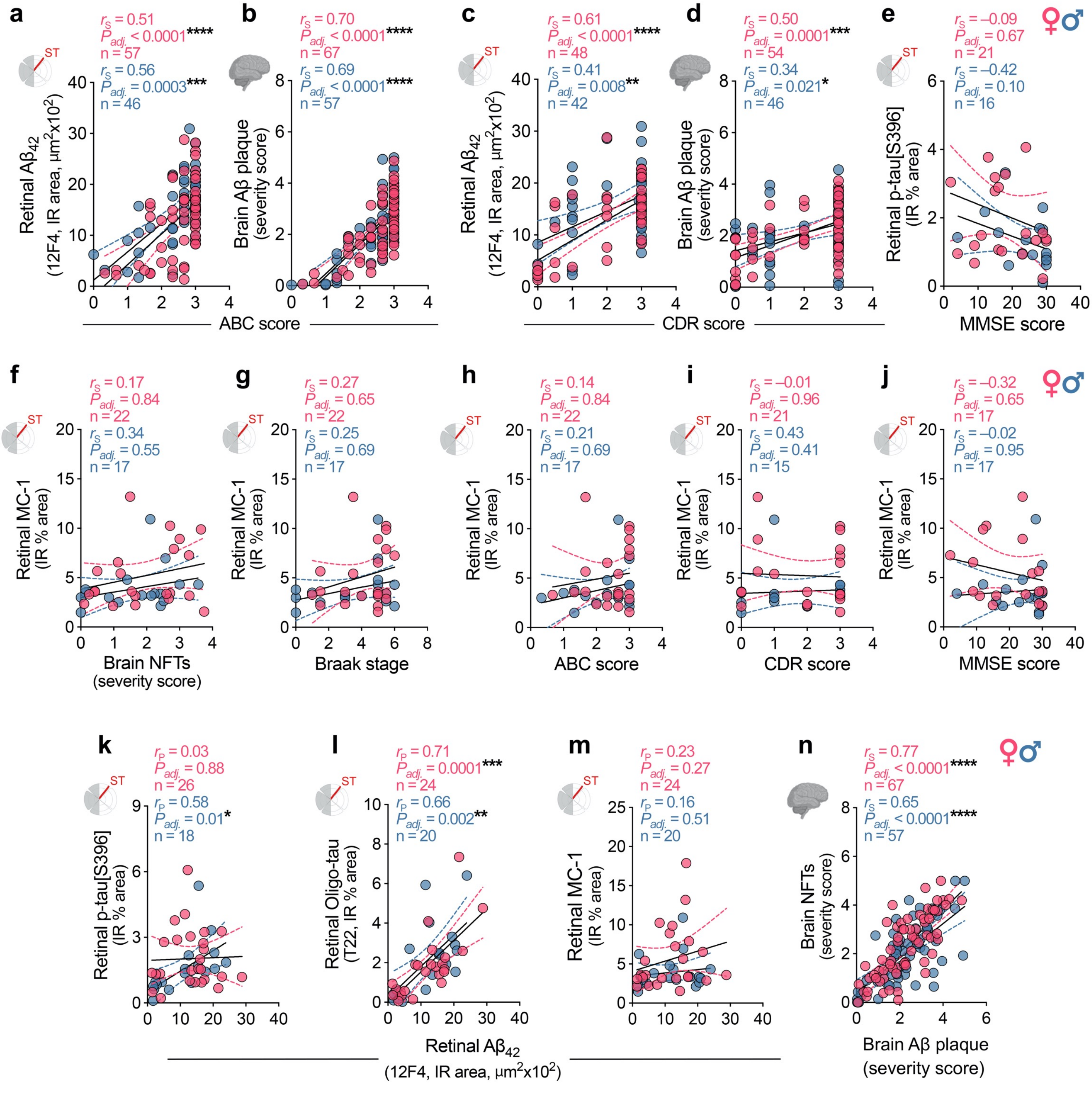

Extended data Fig. 3: Sex-specific associations between retinal Aβ$_{42}$/tau burden, cerebral pathology, disease stage, and cognition. a-d Spearman's correlation analyses between retinal or cerebral Aβ burden with **(a,b)** ABC and **(c,d)** CDR scores. **e** Spearman's correlation analysis between retinal p-tau[S396] and MMSE score. **f-j** Spearman's correlation between retinal MC1$^+$ pretangles and mature tangles IR % area and **(f)** brain NFTs severity score, **(g)** Braak stage, **(h)** ABC score, **(i)** CDR and **(j)** MMSE scores. **k-m** Pearson's correlation analyses between retinal Aβ$_{42}$ and retinal **(k)** p-tau[S396]**, (l)** oligomeric (Oligo)-tau and **(m)** MC1$^+$ pretangles and mature tangles IR % area. **n** Spearman's correlation analysis between brain NFTs severity score and brain Aβ plaque burden. Data are presented as individual values (circles). For each correlation plot, Spearman or Pearson correlation coefficient (*r*), Holm-Šídák adjusted *P* values (asterisks), and number of individuals (n) are shown in the upper right corner. Black lines represent linear regression fits with 95% confidence interval. **P ≤ 0.05, **P ≤ 0.01, ***P ≤ 0.001, ****P ≤ 0.0001, ns: nonsignificant.*

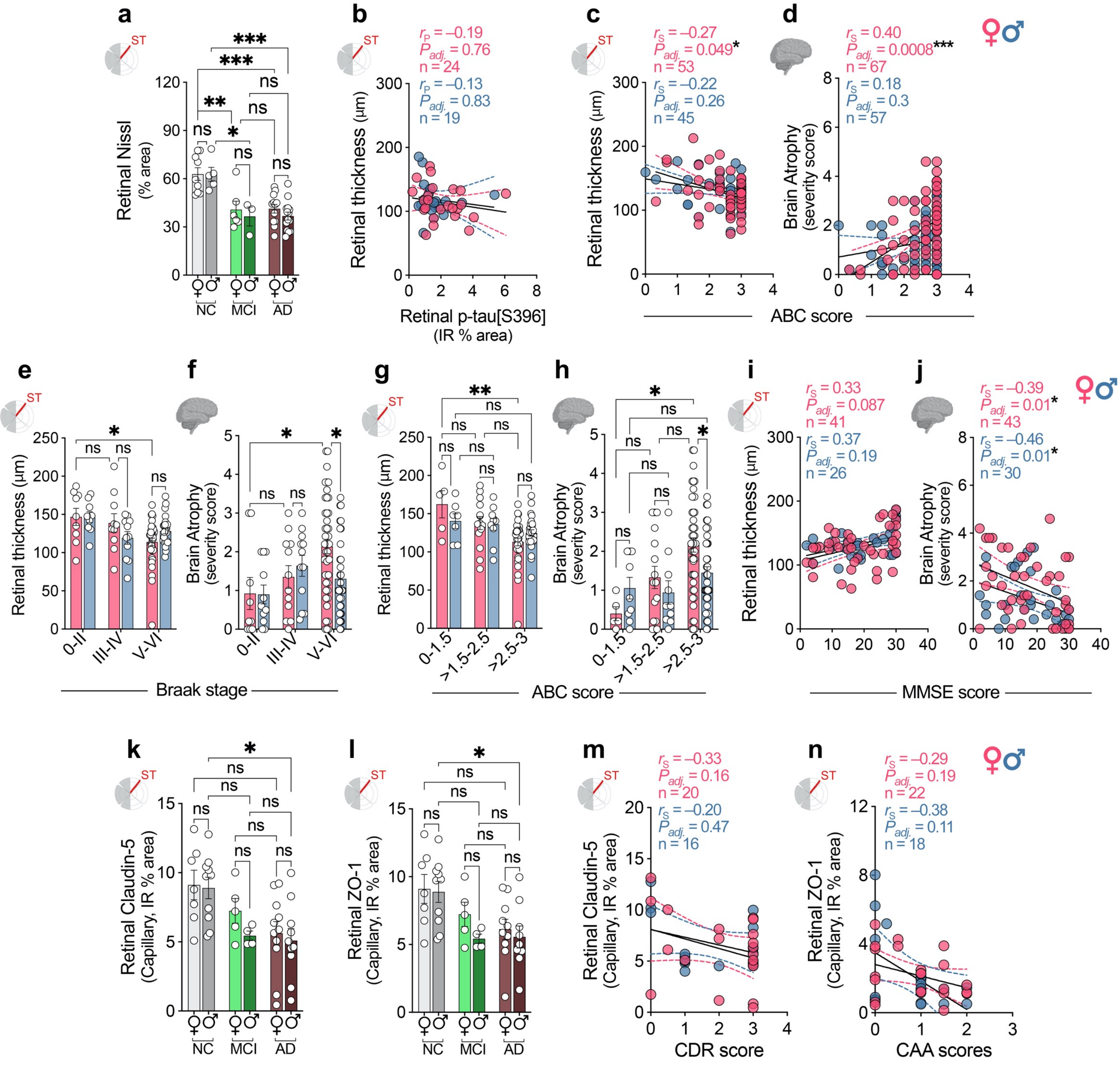

Extended data Fig. 4: Sex-specific associations between neurodegeneration, cerebral pathology, disease stage, and cognition. a Quantitative analysis of Nissl⁺ % area of retinas in a subset of female and male individuals with NC (n=9F/5M), MCI (n=6F/3M) and AD (n= 12F/14M). **b** Pearson's correlation between retinal thickness and p-tau[S396]. **c,d** Spearman's correlation of retinal thickness and brain atrophy with ABC score. **e-j** Retinal thickness and brain atrophy severity score stratified by **(e,f)** Braak stage (retina: n = 9F/9M, 0–II; 10F/11M, III–IV; 34F/25M, V–VI, brain: n = 9F/11M, 0–II; 12F/12M, III–IV; 46F/34M, V–VI) and **(g,h)** ABC score (retina: n = 5F/7M, 0–1.5; 14F/10M, >1.5–2.5; 34F/28M, >2.5–3 brain: n = 5F/9M, 0–1.5; 16F/10M, >1.5–2.5; 46F/38M, >2.5–3). **i,j** Spearman's correlation of retinal thickness and brain atrophy with MMSE score. **k,l** Quantitative analysis of Claudin-5 (NC = 7F/10M, MCI = 5F/4M, AD = 11F/10M) and ZO-1 (NC = 7F/10M, MCI = 5F/4M, AD = 10F/9M) IR % area in females and males across diagnostic groups. **m,n** Spearman's correlation between retinal **(m)** Claudin-5 and CDR score and **(n)** ZO-1 and CAA scores. Data are presented as individual subjects (circles), and group means ± SEMs. Statistical analyses used 2-way ANOVA with Bonferroni's post hoc tests. For each correlation plot, Pearson or Spearman correlation coefficient (*r*), Holm-Šídák adjusted *P* values (asterisks), and number of individuals (n) are shown in the upper right corner. Black lines represent linear regression fits with 95% confidence interval. **P ≤ 0.05, **P ≤ 0.01, ***P ≤ 0.001, ****P ≤ 0.0001, ns: nonsignificant.*

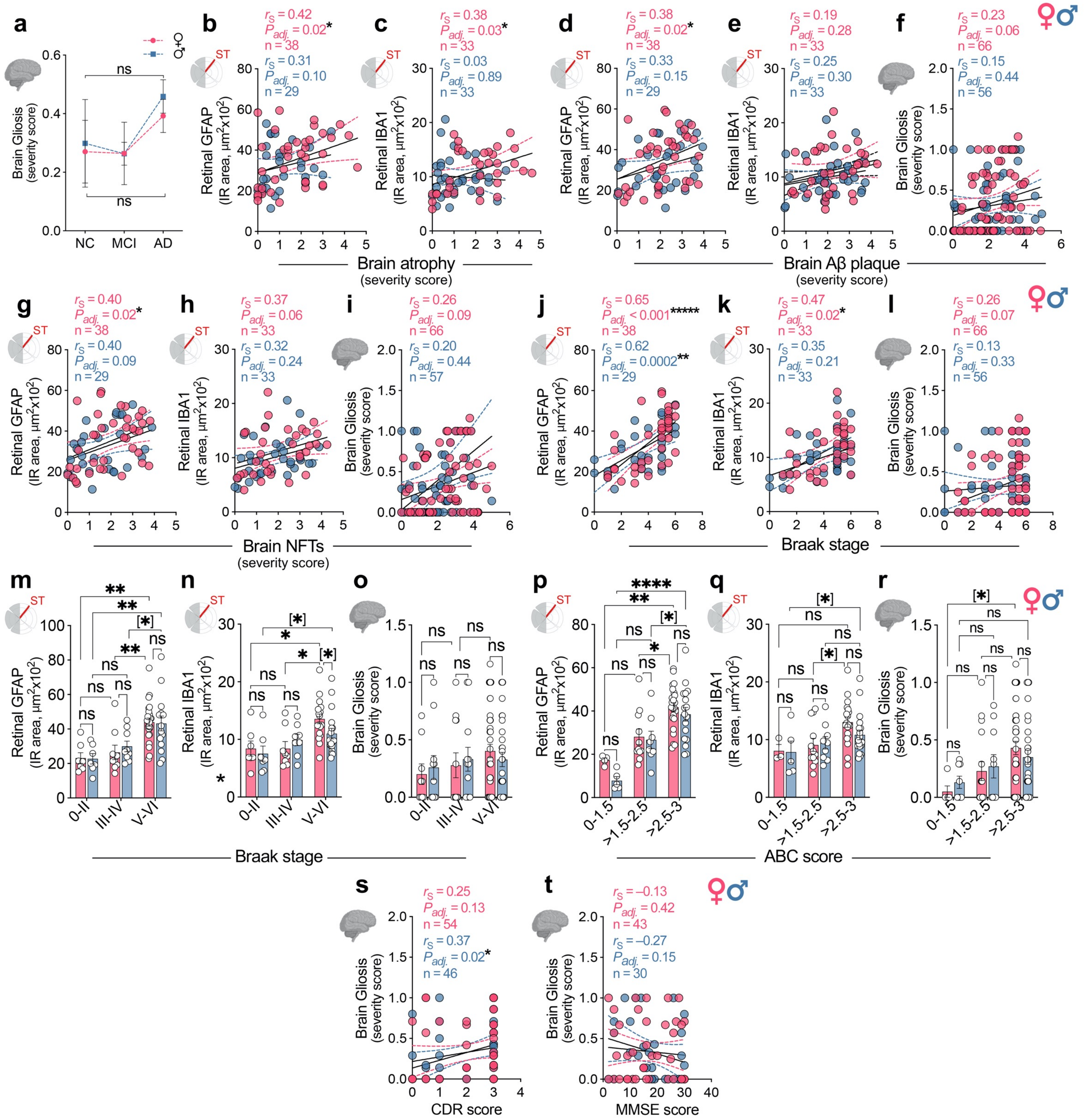

Extended data Fig. 5: Sex-specific stratification and associations of retinal and cerebral gliosis across neuropathological burden, disease stages and cognitive status. a Brain gliosis severity score in MCI, AD and NC individuals. **b,c** Spearman's correlation between retinal GPAF or IBA1 IR area with brain atrophy. **d-l** Spearman's correlation of retinal GPAF, IBA1, cerebral gliosis with **(d-f)** brain Aβ plaque **(g-i)** brain NFTs, and **(j-l)** Braak stage. **m-r** Retinal GFAP+ and IBA1+ immunoreactivity (IR) area, and cerebral gliosis in females and males stratified by **(m-o)** Braak stage (n = 7F/7M, 0–II; 9F/9M, III–IV; 24F/15M, V–VI) and **(p-r)** ABC score (n = 4F/5M, 0–1.5; 11F/8M, >1.5–2.5; 24F/17M, >2.5–3). **s,t** Spearman's correlation between cerebral gliosis and **(s)** CDR score, **(t)** MMSE score. Data are presented as individual subjects (circles), and group means ± SEMs. Statistical analyses used 2-way ANOVA with Bonferroni's post hoc tests for 3- or more group comparisons and 2-tailed unpaired Student's *t*-test for 2-group comparisons (in parentheses). For each correlation plot, Pearson or Spearman correlation coefficient (*r*), Holm-Šídák adjusted *P* values (asterisks), and number of individuals (n) are shown in the upper right corner. Black lines represent linear regression fits with 95% confidence interval. **P ≤ 0.05, **P ≤ 0.01, ***P ≤ 0.001, ****P ≤ 0.0001, ns: nonsignificant.*

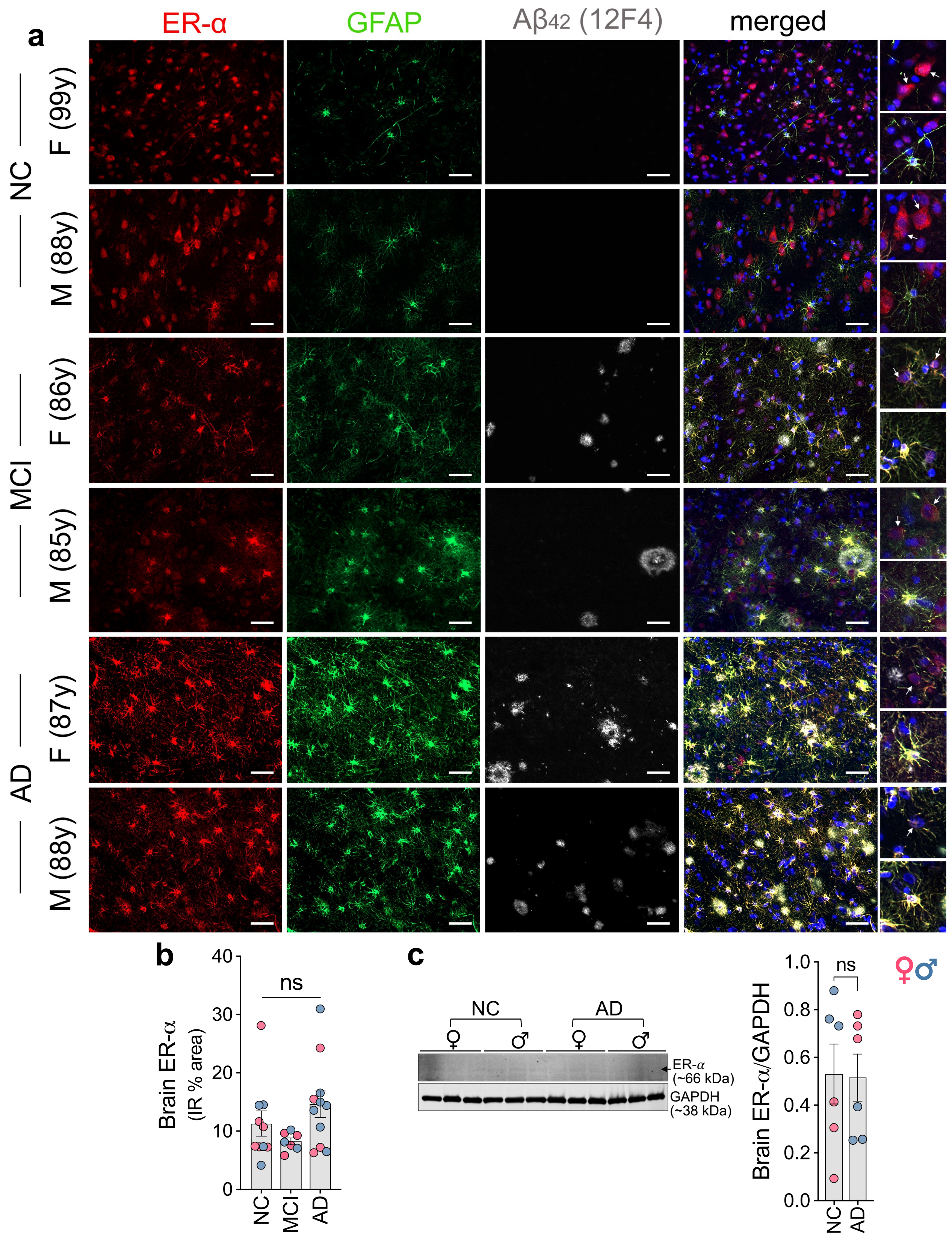

Extended data Fig. 6: Expression of ER-α in the cerebral cortex of NC, MCI and AD individuals. a Representative confocal images of postmortem cerebral cortex sections showing immunolabeling for the ER-α (red), astrocyte marker GFAP (green), and 12F4^{+} Aβ plaque in female and male MCI and AD patient versus NC control. Nuclei are stained with DAPI (blue). Scale bars: 20 µm. 2 repetitions. **b** Quantification of cortical ER-α^{+} immunoreactive (IR) % area across the diagnostic groups (NC=9, MCI=7, AD=10). **c** Representative immunoblots and densitometric analysis of ER-α in cerebral cortex of AD (n=6) versus NC donors (n=6). Data are represented as individual values (circles) and group means ± SEMs. 1-way ANOVA with Tukey's multiple comparison test for 3 or more groups and Student's 2-tailed *t*-test for 2-group comparisons. *ns: nonsignificant.*

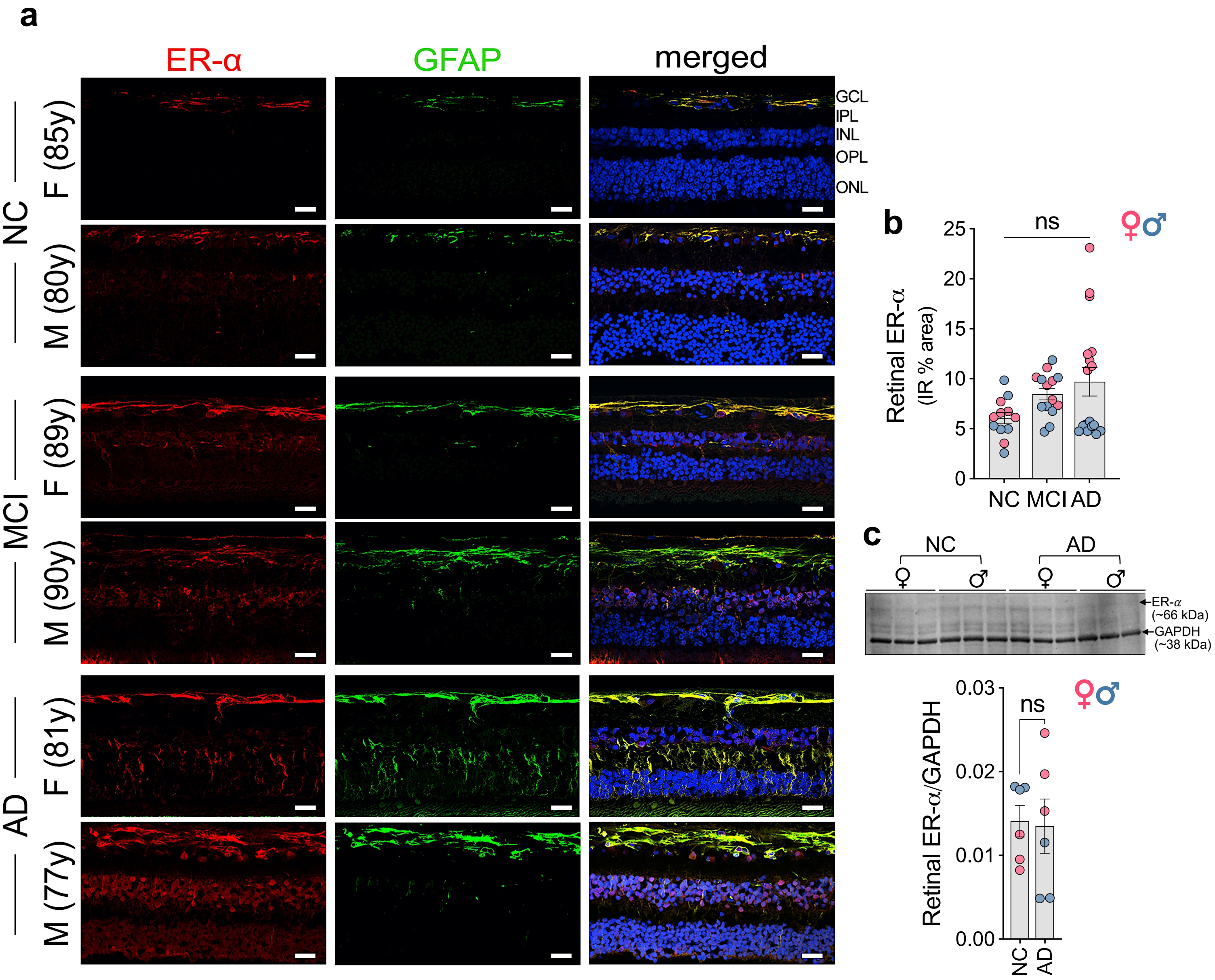

Extended data Fig. 7: Expression of ER-α in the retina of NC, MCI and AD individuals: a Representative confocal images showing immunolabelling for ER-α (red) and GFAP (green, astrocyte and Müller glia), in the postmortem retinal sections from female and male NC, MCI, and AD individuals. Nuclei are stained with DAPI (blue). Scale bars: 20 µm. 3 repetitions. NFL, neurofilament layer; GCL, ganglionic cell layer; INL, inner nuclear layer; OPL, ONL, outer nuclear layer. **b** Quantification of retinal ER-α^{+} IR % areas across the diagnostic groups (NC=15, MCI=17, AD=17). **c** Representative immunoblots and densitometric analysis of ER-α in retina of AD (n=6) versus NC donors (n=6). Data are represented as individual values (circles) and group means ± SEMs. 1-way ANOVA with Tukey's multiple comparison test for 3 or more groups and Student's 2-tailed *t*-test for 2-group comparisons. *ns: nonsignificant.*

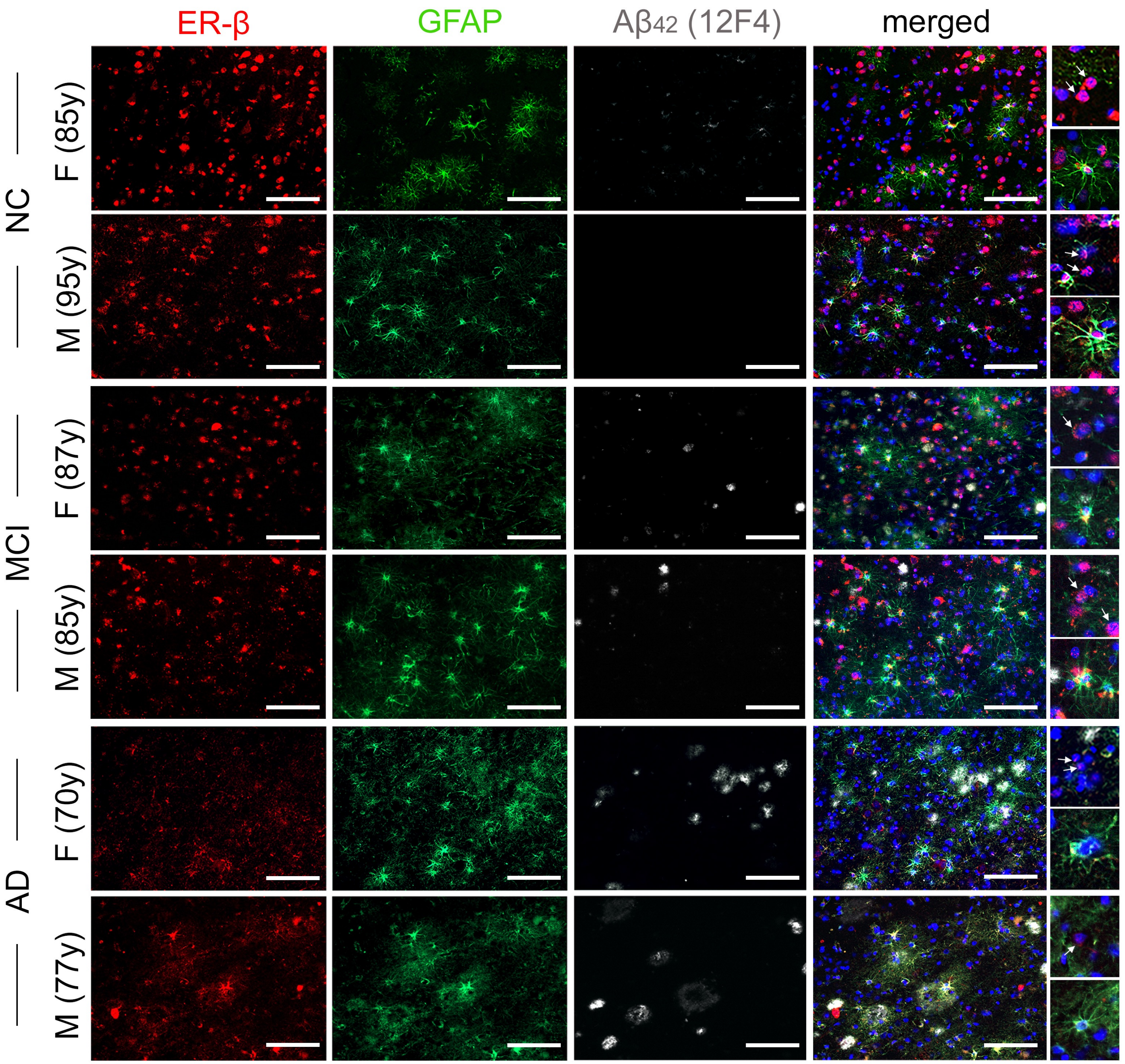

Extended data Fig. 8: Expression of ER-β in the cerebral cortex of NC, MCI and AD individuals. Representative confocal images of postmortem cerebral cortex sections showing immunolabeling for the ER-β (red), astrocyte marker GFAP (green), and $12F4^{+}$ Aβ plaque in female and male MCI and AD patient versus NC control. Nuclei are stained with DAPI (blue). Scale bars: 20 μm. 2 repetitions.

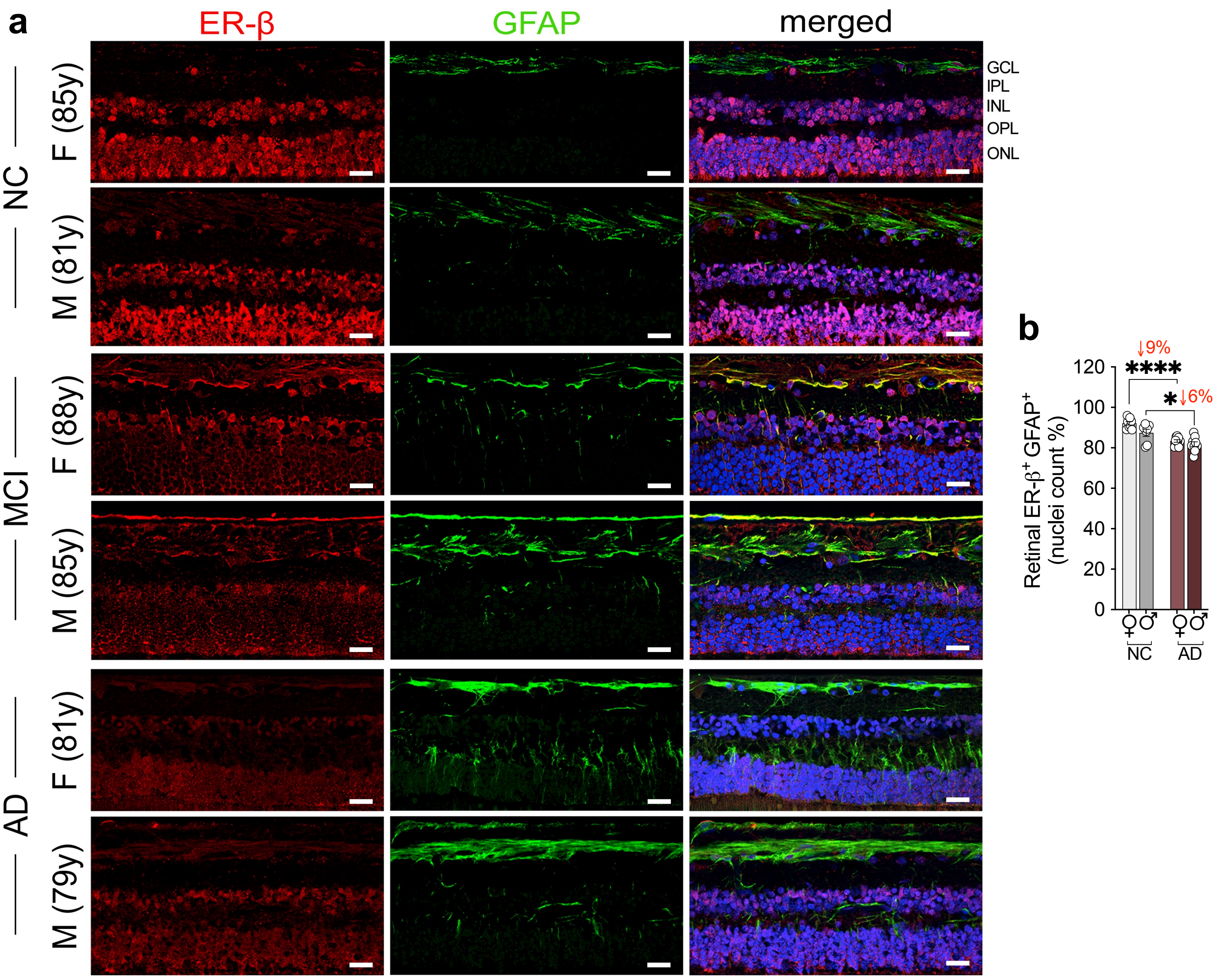

Extended data Fig. 9: Expression of ER-β in the retina of NC, MCI and AD individuals. a Representative confocal images of postmortem retinal sections showing immunolabeling for the ER-β (red) and GFAP (green, astrocyte and Müller glia) in female and male MCI and AD patient versus NC control. Nuclei are stained with DAPI (blue). Scale bars: 20 µm. 3 repetitions. NFL, neurofilament layer; GCL, ganglionic cell layer; INL, inner nuclear layer; OPL, ONL, outer nuclear layer. **b** Quantification of ER-β^{+} GFAP^{+} retinal astrocytic nuclei in female and male AD (n = 9F/9M) and NC (n = 9F/7M) retinas. Percentage change are indicated in red. Data are presented as individual subjects (circles), and group means ± SEMs. Statistical analyses used 2-way ANOVA with Bonferroni's post hoc tests. **P ≤ 0.05, ****P ≤ 0.0001.*

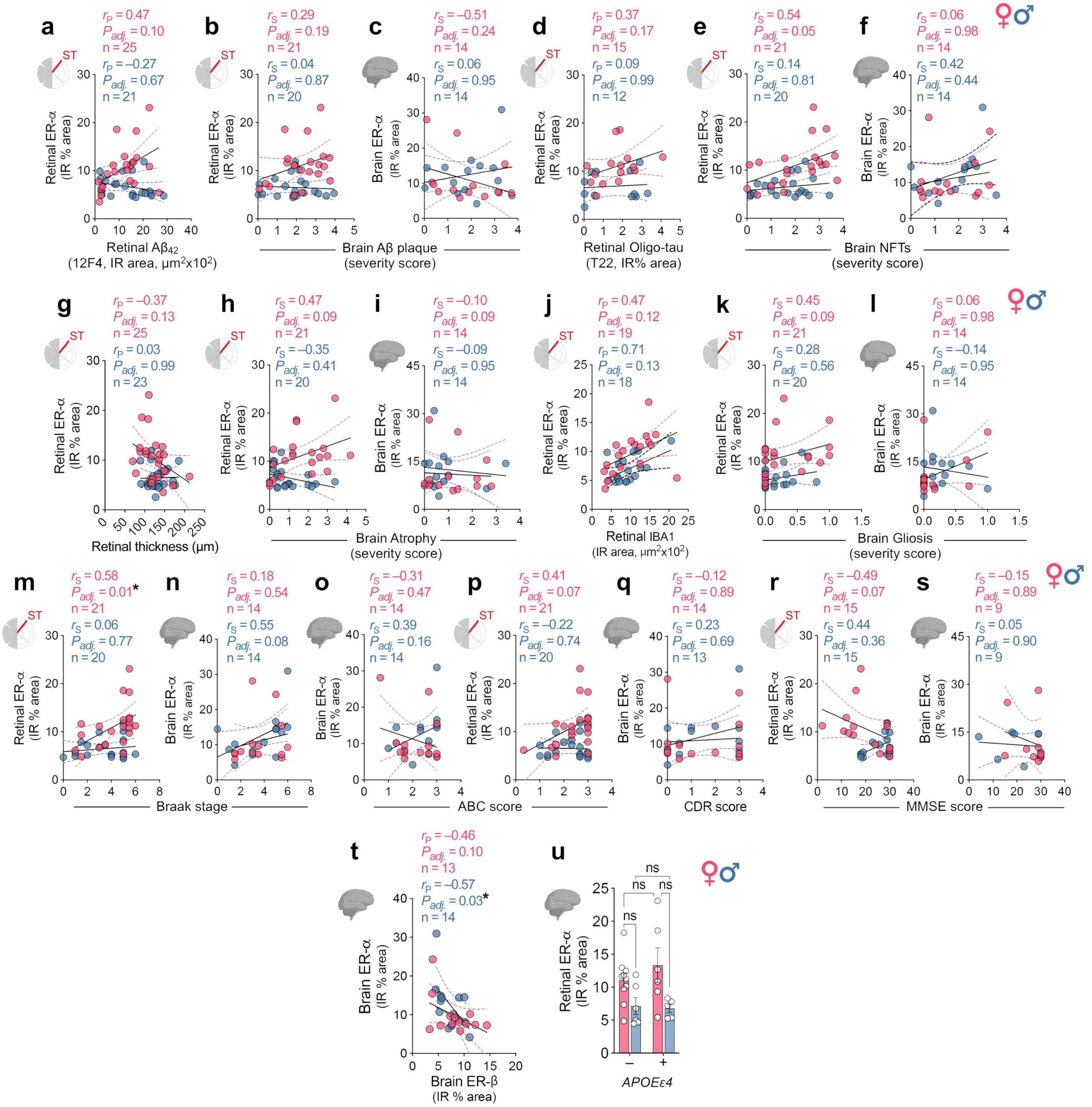

Extended data Fig. 10: Sex-specific associations of retinal and cerebral ER-α with disease stage, cognition, and *APOEε4* genotype. a-t Pearson and Spearman correlation between retinal or cerebral ER-α with **(a-c)** retinal and cerebral Aβ burden, **(d-f)** retinal and cerebral pathological tau burden, **(g-i)** retinal thickness and brain atrophy, **(j-l)** retinal and brain gliosis, **(m,n)** Braak stage, **(o,p)** ABC score, **(q)** CDR score, and **(r,s)** MMSE score. **(t)** Pearson correlation between brain ER-α and ER-β. **u** Sex-specific stratification of retinal ER-α across *APOEε4* carriers and non-carriers (n = 6F/9M ε4 carriers; 13F/9M, non-carriers). Data are presented as individual subjects (circles), and group means ± SEMs. 2-way ANOVA with Bonferroni's multiple comparison test for 3 or more group comparisons. For each correlation plot, Pearson or Spearman correlation coefficient (*r*), Holm-Šídák adjusted *P* values (asterisks), and number of individuals (n) are shown in the upper right corner of each graph. Black lines represent linear regression fits with 95% confidence interval. **P* ≤ 0.05, *ns: nonsignificant.*

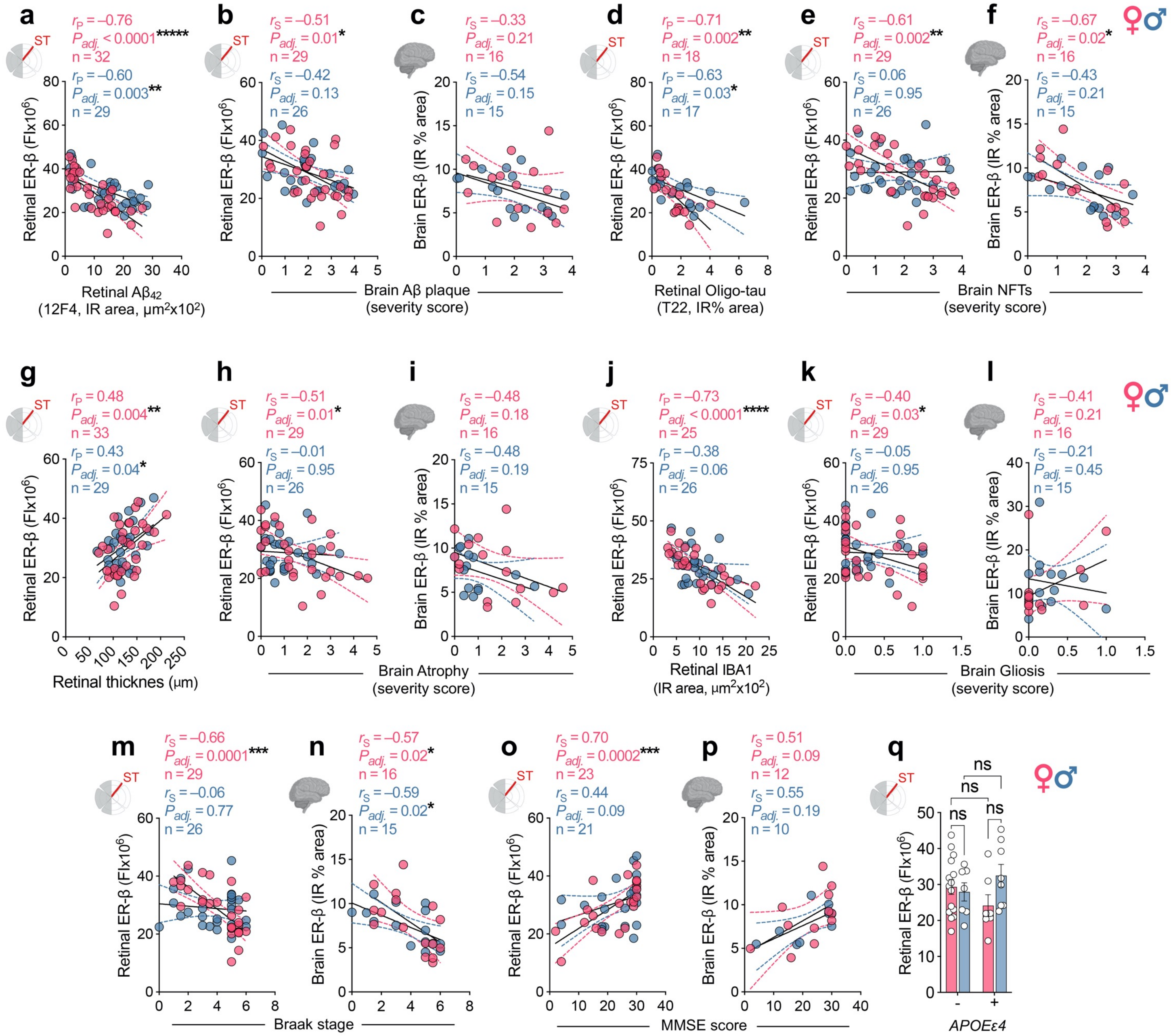

Extended data Fig. 11: Sex-specific associations of retinal and cerebral ER-β with disease stage, cognition, and *APOEε4* genotype. a-p Pearson and Spearman correlation analyses between retinal or cerebral ER-β and **(a-c)** retinal and cerebral Aβ burden, **(d-f)** retinal and cerebral pathological tau burden, **(g-i)** retinal thickness and brain atrophy, **(j-l)** retinal and brain gliosis, **(m,n)** Braak stage, and **(o,p)** MMSE score. **q** Sex-specific stratification of retinal ER-β across *APOEε4* carriers and non-carriers (n = 7F/8M ε4 carriers; 15F/7M, non-carriers). Data are presented as individual subjects (circles), and group means ± SEMs. 2-way ANOVA with Bonferroni's multiple comparison test for 3 or more group comparisons. For each correlation plot, Pearson or Spearman correlation coefficient (*r*), Holm-Šídák adjusted *P* values (asterisks), and number of individuals (n) are shown in the upper right corner of each graph. Black lines represent linear regression fits with 95% confidence interval. **P ≤ 0.05, **P ≤ 0.01, ***P ≤ 0.001, ****P ≤ 0.0001, ns: nonsignificant.*
